# Bekenstein’s Entropy Bound-Particle Horizon Approach to Avoid the Cosmological Singularity

**DOI:** 10.3390/e22070795

**Published:** 2020-07-21

**Authors:** James R. Powell, Rafael Lopez-Mobilia, Richard A. Matzner

**Affiliations:** 1 Department of Physics and Astronomy, University of Texas at San Antonio, San Antonio, TX 78249, USA; Rafael.LopezMobilia@utsa.edu; 2 Theory Group, University of Texas at Austin, Austin, TX 78712, USA; richard.matzner@sbcglobal.net

**Keywords:** Bekenstein entropy bound, cosmological singularity, holographic entropy bound, quantum particle horizon

## Abstract

The cosmological singularity of infinite density, temperature, and spacetime curvature is the classical limit of Friedmann’s general relativity solutions extrapolated to the origin of the standard model of cosmology. Jacob Bekenstein suggests that thermodynamics excludes the possibility of such a singularity in a 1989 paper. We propose a re-examination of his particle horizon approach in the early radiation-dominated universe and verify it as a feasible alternative to the classical inevitability of the singularity. We argue that this minimum-radius particle horizon determined from Bekenstein’s entropy bound, necessarily quantum in nature as a quantum particle horizon (QPH), precludes the singularity, just as quantum mechanics provided the solution for singularities in atomic transitions as radius r→0. An initial radius of zero can never be attained quantum mechanically. This avoids the spacetime singularity, supporting Bekenstein’s assertion that Friedmann models cannot be extrapolated to the very beginning of the universe but only to a boundary that is ‘something like a particle horizon’. The universe may have begun in a bright flash and quantum flux of radiation and particles at a minimum, irreducible quantum particle horizon rather than at the classical mathematical limit and unrealizable state of an infinite singularity.

## 1. Introduction

In a 1989, paper Jacob Bekenstein questions whether the cosmological singularity is thermodynamically possible [1]. In this paper, he reviews four approaches (exotic matter, modifications to classical relativity, quantum field effects, and quantum cosmology) to eliminate the initial singularity from cosmology, concluding that none are satisfactory as they lack information about the microphysics involved and particularly without a full theory of quantum gravity. Bekenstein understood that thermodynamics and his entropy bound specifically as in Equation (1) below [2] might provide insight into the nature of the singularity, stating “Thermodynamics has often been used in such dilemmas, and it is proposed to answer the question of whether there was a Friedmann-like singularity in the universe by exploiting the bound on specific entropy that has been established for a finite system” [1]. Bekenstein first suggested that there is entropy associated with a black hole proportional to the surface area of the black hole, $S_{BH}$ = $\eta k_{B}A/L_{P}^{2}$, where η~1 and $L_{P}$ is the Planck length (1) [3]. (His conjecture became a full-blown theorem with Hawking’s demonstration of black hole temperature and black hole evaporation [4,5].) Remarkably, black hole entropy is additive to “ordinary” entropy, such as the entropy of a hot gas. “The second law is not really transcended provided that it is expressed in a generalized form: The common entropy in the black-hole exterior plus the black-hole entropy never decreases” [6]. Bekenstein later proposed that no closed surface at a particular time could contain more entropy than that calculated for a black hole horizon of the same size, $S\leq k_{B}A/(4l_{P}^{2}$) = $4\pi k_{B}GM^{2}$/(ℏc), where A and M are the area and mass of the black hole [2]. This implies that if a complete physical system with negligible gravity can be enclosed in a sphere of radius *R*, then 2πR/ℏc sets an upper bound on the ratio of the entropy *S* that the system may contain, to its total energy *E*:

(1) 2πR/(ℏc)≥S/E

Here, ℏ is the reduced Planck constant, and *c* is the speed of light.

**Figure 1:**
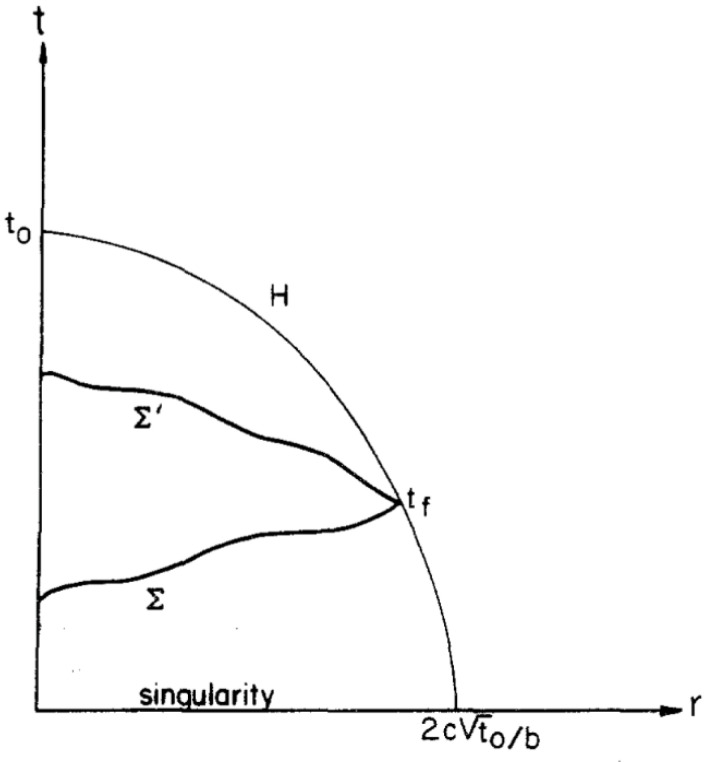
Particle horizon *H* of observer *O* living at time $t_{0}$, and sections of two spacelike hypersurfaces *∑* and *∑*′ entirely within *H* and intersecting it at time $t_{f}$.

Bekenstein [1] applies this to the universe by considering the connected spatial region within the particle horizon of a given observer. The classical definition of the particle horizon is the locus of the most distant points that can be observed by an observer at an event at a specific time *t*_0_ in a cosmology. For a cosmology like the Friedmann models, which are spatially homogeneous and spherically symmetric around each point, this is the 2−D intersection of the past (3−D) light cone with cosmic time t = 0. This coordinate sphere is then taken forward in time as a time-like tube to define a metric sphere with the same coordinate radius at time t = $t_{0}$ (Hardly ever clearly distinguished, many authors, Bekenstein included, also use “particle horizon” to refer to the complete past lightcone of the event at *t*_0_, and their meaning can be determined only by a close inspection of their application in [7,8]. (For more complicated cosmologies, the light cone may become undefined as *t* → 0. Bekenstein dealt with the simple Friedmann case.)). Using an idealized Friedmann cosmological model with effectively massless radiation as its matter source, Bekenstein postulates the metric surface area of the particle horizon to define the quantity R in his limit 2πR/(ℏc)≥S/E. Bekenstein notes that explicitly computing the quantities *S* and *E* in the volume bounded by the past light cone at time *t*_0_ shows that the entropy of radiation in such a region can exceed his entropy bound if the observer is placed too early in the universe; thus, accepting the universality of his entropy bound leads to the conclusion that Friedmann models cannot be extrapolated back to nearer than $t_{0}$~a few Planck times from the initial cosmological singularity (A truism concerning the initial cosmological singularity is that it is a feature of the mathematical model used in the description of a physical system that has no physical relevance since any physical quantity is by definition measurable). (The Planck time $t_{P}={\left(\hbar G/c^{5}\right)}^{1/2}\sim 5.39\times 10^{-44}s$; here, *G* is the gravitational constant.) Thermodynamics thus dictates that an initial Friedmann-like cosmological singularity is physically impossible. Our analysis is based on Bekenstein’s proposal that at a particular time *t*_0_ there is a finite particle horizon coordinate radius $r_{0}$ as depicted in [1] Figure 1, provided as Figure 1 below and in [7]. In Friedmann cosmology, the apparent horizon coordinates radius $r_{0}\to 0$ as $t_{0}\to 0$, and in singularity theorems [8]; however, this analysis shows that there is a time $t_{0}$ before which the universe could not have been Friedmann-like. Hence, we will demonstrate that the backward null cone never reaches t = 0 in an effective Friedmann cosmology but ends at an early time~$t_{P}$. Additionally, the coordinate radius, and thus the metric radius of the apparent horizon at $t_{0}$, is smaller than expected in a classic Friedmann cosmology; and can be represented by a *quantum particle horizon* **given size and thermal blackbody nature**. A particle horizon-like surface at time ~$t_{P}$ is a boundary where, beyond this minimum radius, the Friedmann universe is undefined and not observable.

As suggested by Bekenstein in his paper: “Alternatively, were the universe to materialize out of nothing somewhat after the fashion of Vilenkin’s model [9], we might have a situation where the region visible to an observer is limited in dimension, but is never smaller than some minimum size dictated by the radius of curvature of the emergent spacetime. Here again there would be no singularity but there might be *something like* a particle horizon. Application of the bound on entropy to the appropriate region might then tell us that its dimension cannot be smaller than the Planck length ($l_{P}={\left(G\hbar /c^{3}\right)}^{1/2}$~$1.62\times 10^{-35}$ m) …” [1], which is our suggested quantum-scale particle horizon (to be defined precisely later in Section 3). Furthermore, “With the discovery of the expansion of the universe (Hubble, 1929 [10]) Friedmann’s cosmological models (Friedmann, 1922 [11], 1924 [12]) were shown to be the right framework for description of the recent universe. Can they be extrapolated to the very beginnings of the universe?” [1]. It is not reasonable to expect the current, late-time universe Friedmann model to hold explicitly to a zero-radius beginning.

We propose that our quantum-scale particle horizon at the edge of classical Friedmann models is more reasonable than the desire is to extend Friedmann model validity back beyond the earliest redshift of $z\sim 10^{32}$, the Planck era. Bekenstein continues, “At the end of this cosmic tunnel looms the initial cosmological singularity. It is the consensus in gravitational theory and cosmology that accepting the singularity as a real feature of the universe brings with it thorny issues … no road guarantees excision of the singularity … whatever the mechanism responsible, the universe is loath to begin from a singularity” [1]. The singularity is a mathematical conjecture only. The physical beginning is best characterized by some version of quantum gravity, but consistent with the quantum-size particle horizon suggested here, and consistent with the long-held view that quantum mechanics holds the solution to the cosmological singularity. From Berry, “… The initial singularity is a consequence of the equations of general relativity, and this theory might break down under conditions of very high density and pressure. The most likely possibility is that *quantum effects* (his italics) might occur and prevent localization within infinitely small volumes. (Remember that the first triumph of quantum mechanics was to solve the problem of the electromagnetic collapse of the atom that would be caused by classically radiating electrons spiraling into the nucleus.)” [13].

The purpose of this paper is to propose the Bekenstein particle horizon as a quantum particle horizon alternative to the initial singularity. We look at Friedmann’s model extrapolated to the beginning; formulate an entropy bound-particle horizon following Bekenstein’s suggestion; examine the holographic entropy bound approach to particle horizons; show the thermodynamic impossibility of an infinite singularity; discuss the Wheeler-type information limits of an initial cosmological horizon; and, lastly, summarize the feasibility of the initial quantum particle horizon.

## 2. Friedmann Models and the Particle Horizon of an Early Observer

To address whether Friedmann’s cosmological models can be extrapolated to the very beginning of the universe, we consider the metric of an assumed spatially flat, radiation-dominated Friedmann model at early times from [1] Equation (2). The spacetime interval is:

(2) $$ds^{2}=-c^{2}dt^{2}+a^{2}\left(t\right)\left[dr^{2}+r^{2}\left(d\theta^{2}+sin^{2}\theta d\varphi^{2}\right)\right]$$

where $a^{2}\left(t\right)$ is the scale factor term, substituted for ($b^{2}t$) in Bekenstein’s Equation (2). This time coordinate, t, is called the cosmic time, and the spatial part of the cosmology, $a^{2}\left(t\right)\left[dr^{2}+r^{2}\left(d\theta^{2}+sin^{2}\theta d\varphi^{2}\right)\right]$, is homogeneous 3−D Euclidean space for any particular choice of $t>>t_{P}$. One of the Einstein equations for this cosmology gives the expansion of the universe via its scale factor *a*:

(3) $$a^{-2}{\left(da/dt\right)}^{2}=\left(8\pi G/3\right)\rho_{tot}c^{-2}$$

where $\rho_{tot}$ is the total energy density. (Equation (3) is also called the Friedmann equation). Our assumption that the matter content near t=0 consists of massless radiation implies the usual definitions for energy density $\rho =a_{SB}T^{4}$, where T is the radiation temperature, and for entropy density $s=\left(4/3\right)\;a_{SB}T^{3}$ per species of massless boson, N; and where “N is the effective number of boson particle species present in the early universe” (To refine the factor N in terms of the statistical mechanics of particles, we know that N is not a fixed physical constant, but is at least a function of the physics at various temperatures, so we view it as a slowly varying parameter depending on T. For instance, Weinberg gives N=10.75, considering spin states or spin degeneracy [14], Equation (3.1.15). This number experimentally holds to temperatures $\sim 10^{11}\;K$ approximately 0.01 s after the beginning in the standard model, or $\sim 10^{12}\;K$ at ~0.0001 s [15]. If we extrapolate beyond the limit of known particle physics (near the quark-gluon plasma temperature of 10^12^ K [16] to $T_{P}\sim 10^{32}\;K$, this all becomes speculative, but we reasonably assume that the number of effectively massless particles is never very large, but possibly >> 1 at some time in the early universe. We argue below that N→1 as $T\to T_{P}\sim 10^{32}\;K$.) (Here, $a_{SB}$ is the Stefan-Boltzmann constant.) Hence, $\rho_{tot}=N\rho$. Simple expanding Friedmann cosmology allows for the straightforward calculation of entropy density and energy density at any time. Because this cosmology is spatially homogeneous if the correct cosmic time coordinate is used, those qualities depend only on that cosmic time. The entropy density scales as the third power of a temperature and we know that in these Friedmann cosmologies the temperature redshifts proportional to $a^{-1}$, so entropy scales as $a^{-3}$. A different scaling law holds for the energy density; it is proportional to the fourth power of the temperature so energy density scales as $a^{-4}$. Because the volume of any portion of space defined by given spatial coordinates increases as $a^{3}$ in this cosmology, it is easy to see that (*total entropy in such a volume*) = (*entropy density* × *volume*) remains constant; thus, in any particular region, entropy is conserved. On the other hand, because energy density scales as $a^{-4}$, the total energy in a particular volume scales as $a^{-1}$. A consequence of $\rho_{TOT}$ ∝ $a^{-4}$ in Equation (3) is that the expansion factor is:

(4) $$a=bt^{1/2}$$

The singularity (a=0) is at t=0 for all space points. (Here, b is a constant addressed later.) These relationships lead to our derivation of temperature (modulo factors of order unity) as

(5) $$T=c^{5/4}\;\hbar^{3/4}{\left(GN\right)}^{-1/4}t^{-1/2}=N^{-1/4}\;\hbar {\left(t_{0}t_{P}\right)}^{-1/2}$$

where $t_{P}$ is the Planck time and $t_{0}$ is a particular specific time (after the singularity) at which the temperature is evaluated. The backward light cone from an event at that particular time $t_{0}$ in this Friedmann cosmology is found by integrating the condition $ds^{2}=0$ for relativistic, ‘probably massless’ light-like particles. The resulting null 3-surface H, as shown in Figure 1 below, is written in parameterized form as a Bekenstein hypersurface at time t:

(6) $$t={\left[t_{0}^{1/2}-br/\left(2c\right)\right]}^{2}$$

As stated in Ref. [1] Equation (3). At a particular time, t, this defines a spatial 2−sphere and gives its coordinate radius, r. If we set t=0, we obtain the coordinate radius $r_{0}=2ct_{0}^{1/2}/b,$ Figure 1, which shows how far in coordinate terms from r = 0 a signal may start at t = 0 and hope to reach an observer O at the origin at $t=t_{0}$. “The hypersurface (6), which encloses the part of the universe visible to O is therefore called the particle horizon of O” [1]; it is the maximum coordinate distance that an observer at a particular time *t*_0_ can see.

We note here that the Bekenstein calculation of the entropy/energy ratio is “the largest (S/E) observable to O” with ‘O’ being a “single fiducial observer” [1]. He computes this ratio (S/E) in Friedmann cosmology by integrating on spacelike surfaces (e.g., $t=t_{f}$ in Figure 1) that intersect the lightcone that runs into the past from a fiducial event at time $t=t_{0}$. (Slices like *∑* or *∑*’ in Figure 1 give the same total entropy because, as noted above, the total entropy within a given boundary is conserved, i.e., independent of time, but *∑*′ gives smaller total energy because of the falloff of total energy with time, in a volume with a fixed spatial boundary.)

Bekenstein computed the integrals of r and s over bounded spacelike hypersurfaces such as *∑* and *∑*′ in Figure 1. Because $S/E\sim \left(E/T\right)/E\sim T^{-1}$, these ‘slices’ are dominated by late times ($\to t_{0}$) because temperature decreases with time. Thus, near $t_{0}$ (again modulo constants of order unity),

(7) $$S/E=1/T={\left[G/\left(\hbar^{3}c^{5}\right)\right]}^{1/4}N^{1/4}t_{0}^{1/2}=\;N^{1/4}{\left(t_{0}t_{P}\right)}^{1/2}/\hbar$$

From Ref. [1] Equation (14). To write the bound on (S/E) we need to define the sphere bounding the region with large (S/E). Bekenstein chooses the metric radius R of the particle horizon at $t=t_{0}$. Not surprisingly, this evaluates to $R=2ct_{0}$, so Bekenstein’s bound now reads:

(8) $$2\pi R/\left(\hbar c\right)=4\pi ct_{0}/\left(\hbar c\right)=4\pi t_{0}/\hbar \sim t_{0}/\hbar \;\geq \;S/E=N^{1/4}{\left(t_{0}t_{P}\right)}^{1/2}/\hbar$$

We simplify this bound further to:

(9) $$1\geq N^{1/4}\;{\left(t_{P}/t_{0}\right)}^{1/2}$$

With our assumption that N is a moderately small number (see note above after Equation (3)), $N^{1/4}$ is of order unity, so we shall drop the explicit appearance of N subsequently, which is justified more fully below. We conclude that the constraint for time, then, is that t≥ ($G\hbar /c^{5}$)^1/2^ = $t_{P}$ for Friedmann equation consistency with entropy bounds. Spacetime becomes classically undefined earlier than this time; we can go no earlier than $t_{P}$ because the Friedmann model becomes inapplicable. At time $t_{P}$, our Equation (7) becomes an equation in terms of the Planck temperature:

(10) $${\left(S/E\right)}_{t_{P}}=1/T_{P}$$

The Planck temperature which occurs at the Planck scale can be considered the maximum temperature of the universe as suggested by Sakharov, $T_{max}=k_{B}^{-1}c^{5/2}\hbar^{1/2}G^{-1/2}=1.42\times 10^{32}$ degrees [17].

## 3. An Entropy Bound-Particle Horizon Precluding the Cosmological Singularity

From the radiation-dominated Friedmann model, Bekenstein showed how there is a contradiction with his entropy bound if the model is extrapolated too far back in time, and there is even the suggestion that the physics of quantum gravity prevents the singularity [1]. The classical definition of particle horizon is the intersection of the past light cone with the initial singular surface at t=0. With Bekenstein’s entropy bound suggesting an earliest time admitting a semiclassical description at $t\sim t_{P}$, we define a quantum particle horizon, which is the coordinate sphere defining the past light cone at $t=t_{P}$. This sphere has a smaller coordinate radius than the classical particle horizon (In our coordinate radius case, the r of the apparent horizon at $t=t_{min}\sim t_{P}$ is cf. Equation (6) (above): $r=2c\left(t_{0}^{1/2}-t_{min}^{1/2}\right)/b$. Then, from Equation (2), the metric radius *R_H_* of the apparent horizon evaluated at t = $t_{0}$ is *R_H_* = $brt_{0}^{1/2}$ = $2ct_{0}^{1/2}\left(t_{0}^{1/2}-t_{min}^{1/2}\right)$. Setting $t_{min}=t_{P}$ shows that, for $t_{0}>t_{P}$, this $R_{H}$ is smaller than that computed with a null cone that goes all the way to *t* = 0.) but has nonzero metric radius~$l_{P}$ at $t=t_{P}$, while the classical particle horizon has a vanishing metric radius at t=0. **The quantum particle nature of the beginning of the universe defines this horizon**. In the Friedmann metric Equation (1), there appears the constant ‘*b*’ with dimension [$t^{-1/2}$], which defines the unit of time. We choose $b^{2}=t_{P}^{-1}$. At the Planck time $t_{P}$, the Hubble parameter H~ $t_{P}^{-1}$. So, the metric takes the form:

(11) $$ds^{2}=-c^{2}dt^{2}+\left(\frac{t}{t_{P}}\right)\left[dr^{2}+r^{2}\left(d\theta^{2}+sin^{2}\theta d\varphi^{2}\right)\right]$$

which allows for an easy inspection of the behavior near $t=t_{P}$. If we consider that the deeper significance of the constant b at this scale may be quantum in nature, we can make the connection to blackbody radiation and Planck’s constant at this quantum-thermodynamic limit of general relativity. To associate a minimum radius r to quantum blackbody radiation released at a particle horizon of this radius, we have already seen how there is a maximum temperature of the universe, and this limiting temperature is the Planck temperature $T_{P}$, Equation (10). By convention, we work in natural units; any temperature, e.g., $T_{P}$ is an energy, with associated length $GT_{P}/c^{4}=l_{P}$, the Planck length. With the assumption that the initial state is coherent, not squeezed (see [18,19]), Heisenberg uncertainty specifies a minimum radius greater than zero in the early universe. For massless bosons in one dimension, we can expand $\Delta p_{x}\;\Delta x\sim \hbar$ with x→r, and, neglecting factors of 2π etc., we restate Heisenberg uncertainty in terms of *r*:

(12) $$\Delta p_{r}\Delta r=\Delta r\Delta \left(\hbar /\lambda \right)=\Delta r\left[-\hbar \left(\Delta \lambda /\lambda^{2}\right)\right]\sim \hbar$$

This means that $\left|\Delta r\Delta \lambda \right|\sim \lambda^{2}$. Assuming both variances are the same (the “*not-squeezed*” assumption), then Δr~λ and Δλ~λ. We can interpret Δr near the beginning then as $r_{min}\sim l_{P}\sim GT_{P}/c^{4}$. Thus, we conclude $r_{min}\sim l_{P}$, and $\lambda \sim l_{P}$, and therefore $r_{min}\sim \lambda =r_{QPH}$, which is the quantum particle horizon radius, and a minimum here at the time of transition from quantum to classical behavior. This connects the large-scale Friedmann model at its minimum radius limit to a quantum initial wavelength where blackbody radiation is initiated at a quantum particle horizon.

## 4. The Holographic Entropy Bound and Particle Horizon Approach

To support a minimum radius precluding the singularity, and since the minimum particle horizon where the entropy bound is contradicted is a spherical surface area, the holographic entropy bound can be directly applied to the particle horizon approach.

The holographic approach to cosmology originated in a 1998 paper by Fischler and Susskind in which they state, based on the holographic principle, “… the entropy of a region must not exceed its (enclosing horizon) surface area in Planck units” [20]. As Diaz, Per, and Segui comment in referring to Fischler and Susskind, “William (Willy) Fischler and Leonard Susskind proposed a cosmological holographic principle based on the particle horizon as an alternative form of the entropy bound:

(13) $$S_{PH}\leq A_{PH}/\left(4l_{P}^{2}\right)$$

$S_{PH}$ is the holographic entropy at the particle horizon, and $A_{PH}$ is the surface area of the particle horizon. The entropy content inside the particle horizon of a cosmological observer cannot be greater than one quarter of the horizon area in Planck units” [21]. Equation (13) is the same relation as the Bekenstein entropy bound of a Schwarzschild black hole. We suggest that the reference to Planck scale units here takes us naturally to the Planck era of cosmology, where the area of the particle horizon approaches the Planck area as $A_{PH}\to l_{P}^{2}$. The holographic version of the entropy bound is violated when $S_{PH}>A_{PH}/\left(4l_{P}^{2}\right)$ at our minimum particle horizon radius $R_{PH}\sim l_{P}=r_{min}$ (as above after Equation (12)), thus ‘holding off’ the singularity at this radius; this supports Bekenstein’s approach with ‘something like a particle horizon’ to prevent the singularity [1]. Again, according to Fischler and Susskind: “Remarkably … The entropy in the universe is as large as it can be without the holographic principle having been violated in the early universe!” [20]. Equation (13) taken to the Planck scale is supported by Diaz et al.: “(The Fischler and Susskind) proposal is the enforcement of the intersection time near the Planck time; thus, the apparent violation of the holographic prescription will be restricted at the Planck era” [21]. They also state “concretely, the FS (Fischler-Susskind) prescription gives us a limit on the entropy density at the Planck time… This fact is usually skipped in the literature”. The result is that both Bekenstein and holographic entropy bounds specify a minimum particle horizon radius approaching the Planck length as we suggest from Bekenstein [1], and a remarkably small amount of entropy within that horizon. The size of the apparent horizon at $t=t_{P}$ is of order the Planck length $l_{P}$, with area $A_{P}=4\pi \;{\left(l_{P}\right)}^{2}$; the Fischler-Susskind entropy bound is then:

(14) $$A_{P}/\left(4l_{P}^{2}\right)=\pi$$

This remarkably small limit on the entropy, which is discussed more fully below in terms of information, strongly suggests that N→1 as $t\to t_{P}$, justifying our assertion of the smallness of N.

In his seminal work on the holographic principle, Bousso compares Bekenstein and holographic entropy bounds [22]. He writes that from his Equation (2.9) ($S_{matter}\leq 2\pi ER$, or in full, $S_{matter}\leq 2\pi k_{B}ER/\left(\hbar c\right)$), which is the Bekenstein bound, one thus obtains $S\leq 2\pi MR\leq \pi R^{2}=A/4$, Equation (2.25) [22], just as in Equation (13). From this, we conclude that the Bekenstein and holographic entropy bounds both result in $r_{min}\sim$ $l_{P}$ > 0 near the beginning.

## 5. Mathematical Conjecture and the Impossibility of an Infinite Singularity

Bekenstein’s search for the non-singular, discrete quantum-nature particle horizon at the beginning may come from his advisor John Wheeler in his desire to avoid singularities. In a recent biographical memoir of Wheeler, Kip Thorne reflects, “John was highly skeptical of the Oppenheimer-Snyder conclusions about the collapse (of a star into a black hole). He focused particularly on the singularity (with infinite density and infinite curvature of spacetime) predicted to form deep inside the cut-off sphere (inside what today we call the event horizon). There, he argued, the laws of classical general relativity must break down, and be replaced by laws of quantum gravity that result from “a fiery marriage” of general relativity with quantum theory…” [23]. In June 1958, at a Solvay Congress [24], Wheeler rejected the predicted singularity as physically unreasonable and speculated about the collapse’s true final state: “… no escape is apparent except to assume that the nucleons at the center of a highly compressed mass [where the singularity is trying to form] must necessarily dissolve away into radiation … at such a rate or in such numbers as to keep the total number of nucleons from exceeding a certain critical number…” [23]. Although Thorne and Wheeler refer here to the gravitational singularity predicted within a black hole, the mathematical limit of infinite density and space-time curvature is the same as for the cosmological singularity. As an aside, it is interesting to note again that since singularities in general relativity involve infinities, some physical quantity, e.g., energy density increasing without limit, it may be that the gravitational singularity where r→0 may also be unreachable in a black hole. There may be quantum particle horizons preventing the existence of every mathematically singular point. No naked singularities would exist, and Penrose’s cosmic censorship hypothesis would be valid without the need of event horizons hiding the singularities [25]. It may be that, rather than no horizon at r=0, naked singularities may not be observed since physical singularities do not exist.

As Wheeler rejected the predicted singularity as a physically unrealizable mathematical abstraction, and argued that the energy density where the singularity is trying to form must necessarily dissolve away into radiation, his analysis presages the function of a quantum particle horizon as the source of radiation and particles at the beginning, also with no cosmological singularity. The source at the minimum radius of this horizon on the order of the Planck length as we propose above and originally proposed by Bekenstein, would be thermal blackbody radiation~Planck wavelength and temperature. This is our proposed quantum particle horizon of photons and relativistic particles around the hypothetical mathematical singularity, preventing its physical materialization.

An initial ‘quantized’ particle horizon as a quantum hold-off radius avoids dependence on the unphysical mathematical conjecture of an infinite cosmological singularity. From Ellis [26], “David Hilbert famously argued that infinity cannot exist in physical reality. The consequence of this statement—still under debate today—has far-reaching implications”. And from Hilbert himself in [27], as quoted by Ellis, “The infinite is nowhere to be found in reality, no matter what experiences, observations, and knowledge are appealed to.” [26]. Ellis goes on to say that just because we have a symbol (∞) to represent infinity does not mean it is part of the natural order, does not prove that zero (such as in r=0) can exist in physical reality either. As he states, “… we can interpret ‘0’ as existence in spacetime of absolutely nothing—which we know not to be the case following the uncertainty principle of quantum mechanics …” [26].

Another alternative to the initial singularity suggested by Bekenstein [1] that we take up here is ‘cosmic bounce’. From Ohanian and Ruffini, “One possible explanation is to suppose that the present expanding universe was preceded by a contracting universe, which passed through a moment of greatest contraction (as in Figure 9.13 [28]). Such a behavior of (scale factor) a(t) … (means) a(t) must remain finite at the moment of maximum contraction, that is, there must be no singularity.” [28]; see also [29]. If there is a moment of maximum contraction, then there is a peak energy density at some minimum radius that cannot be exceeded, whether from a previous universe contraction and bounce, or from an initial release of radiation. Ohanian and Ruffini continue, “The absence of a singularity at the first moment of the Big Bang would seem to be in contradiction with general singularity theorems that have been proven for the solutions of Einstein’s equations. But these theorems deal only with the classical regime; they can be circumvented in the very early universe, where matter is in the form of quantum fields, and where even the geometry is quantized. Furthermore, if the solution of the Einstein equations develops a singularity, this merely indicates a breakdown in the equations, not necessarily an actual singularity in the real world… it is natural to seek some modifications of the equations that avoids the singularity, … (Brandenberger, 1985 [30], 1992 [31]). Thus, the question of whether there is an actual singularity at the first moment of the Big Bang still remains open.” [28]. See also [32,33].

## 6. Inaccessibility of Information Beyond the Planck Scale Quantum Particle Horizon

The Planck-scale cosmological particle horizon derived above in Section 3 is based on the Bekenstein entropy bound, and the connection between entropy and information loss may elucidate this boundary. As we have seen (cf. Equation (14)), at very small scales, $\sim l_{P}$, $S\leq 4\pi l_{P}^{2}/\left(4l_{P}^{2}\right)\sim \pi \;!$ Low entropy near the beginning with corresponding low information loss is described by Penrose as the ‘low entropy conundrum’ and discussed in many of his books and papers [25,34,35]. Low entropy near the beginning is still a mystery—why is entropy so low in the very early universe? (Or perhaps entropy is undefined in quantum gravity situations.) High entropy in the observable universe now means a large information loss since the low entropy beginning; and information loss and entropy are also characteristics of black hole thermodynamics. Extrapolating high entropy and information loss now back to the very early universe points to a thermodynamic information limit near the classically assumed cosmological singularity. To compare high entropy and corresponding information loss in the observable universe now, with the low initial entropy, Egan and Lineweaver estimated the bound on the entropy of the observable universe from the current cosmic event horizon:

(15) $$S_{ceh}=\left(2.6\pm 0.3\right)\times 10^{122}k_{B},$$

As cited in ref. [36] Equation (40), in Boltzmann units. They estimate the total entropy *within* the universe ($1.2\times 10^{103}k_{B})$, almost all of which is from supermassive black holes; clearly, the universe has ample headroom within the entropy bound. Egan and Lineweaver note, “this is the reason (that) dissipative processes are ongoing.” Increasing entropy at the expanding cosmic event horizon here from Egan and Lineweaver is in contrast to the beginning where the Bekenstein and holographic bounds specify a minimum radius. Since $S=k_{B}\ln\Omega$ from (15) an estimate of the possible number of microstates in the observable universe now is $\exp\left[(2.6\pm 0.3)\times 10^{122}\right]\approx 10^{10^{122}}$. This is the very large increase in the number of microstates (very large information loss) at the cosmic event horizon since the beginning. Can this information be recovered, or this vast quantity of information be reassembled? The probability of obtaining the original configuration of microstates at $t_{min}$ near the unique state $\Omega_{i}$ ≈ 1 is $10^{-10^{122}}$ and very unlikely. Egan and Lineweaver point out that cosmic microwave background (CMB) photons are the most significant non-black hole contributors to the entropy of the observable universe. They estimate the size of the observable universe, $R_{\mathrm{obs}}\sim 47\;\mathrm{Glyr}$, and compute its volume assuming the flat 3-space formula $4\pi R^{3}/3$. Equation (13) uses the standard expression for the photon entropy which results in $S_{\gamma }\sim 2\times 10^{89}k_{B}$ [36].

A similar computation had already been done in 1989 by John Wheeler. Wheeler proposed his radical new idea that all physical reality is fundamentally information, which he termed “It from Bit” [37]. Wheeler computed the total CMB entropy as an example of the dissipation, or loss of information, since the “primordial cosmic fireball”. He assumed a 3-sphere (volume $2\pi^{2}R^{3}$) and a smaller radius (R~13 Glyr), and obtained a result similar to Egan and Lineweaver’s. Wheeler’s intent was to substantiate “It from Bit”; modulo a factor of order unity, Wheeler’s number of bits equals the dimensionless entropy. Here, we replicate Wheeler’s calculation,

(16) $$\begin{array}{ll} N\;bits=\left(\log_{2}e\right) & \times \left(number\;of\;nats\right);\;‘nats’=natural\;logarithms\\ & =\left(\log_{2}e\right)\times \left(entropy/Boltzmann’s\;constant,\;k_{B}\right)\\ & =1.44\ldots \times \left[\left(8\pi^{4}/45\right){\left(radius\times k_{B}T/\hbar c\right)}^{3}\right]=8\times 10^{88}\;bits \end{array}$$

Ref. [37]. Wheeler equates this to the number of bits of information needed to create the CMB we now observe, from the primordial cosmic fireball that we cannot observe, quantifying the increase in its entropy. Since ΔS=−ΔI [6,38], this loss of access to the original information is −(8 × 10^88^) bits, which is a measure of the irretrievability of this number of bits of information 2 × $10^{89}$ above.

The thermodynamic nature of the *spacelike* particle horizon near the cosmological singularity may also hint at how to solve the information loss paradox and the conflict with unitarity in quantum mechanics. From this, we suggest that information may not be destroyed *absolutely* in the *space* containing the singularity behind the event horizon of a black hole, or over *time* since $t_{min}$ near the beginning (both taken together as *spacetime*), but it has disappeared *observationally*. Information is continually being lost in all natural time-dependent processes due to the second law of thermodynamics, and is not recoverable now in our current spacetime reference frame. Being ‘lost’ to observation does not necessarily mean destruction of the information, nor that information content is fundamentally erased—it is just inaccessible—which could resolve the conflict with unitarity. This idea is consistent with the Penrose cosmic censorship conjecture [25], where naked singularities exist mathematically as solutions to Einstein’s equations, but may not form or be found in the real world. For black holes, this means that the gravitational collapse of a (non-singular) mass never results in a naked singularity in spacetime that might be observed, but rather as a mathematical singular point hidden behind an event horizon; postulated, but not observable. On the other hand, we conclude that, at the cosmic level in the very early universe, the cosmological singularity effectively does not exist beyond $r_{min}$, where spacetime is unobservable and thus undefined.

## 7. Summary of Results and Conclusions

In the standard big bang model of cosmology, before inflation, the universe began with a burst of high-energy thermal radiation. As Penrose states, “The very early universe was a thermal state … the 2.7 K microwave background radiation that represents the actual ‘flash’ of the Big Bang, (is) still in evidence today, …” [25]. We conclude that this thermal blackbody radiation of photons and relativistic particles at the beginning, observed now in the cosmic microwave background, could never have had a wavelength of zero by definition. Additionally, we conclude that the initial particle horizon radius must also have been non-zero, as a consequence of Heisenberg’s Uncertainty Principle, setting a limit beyond which Friedmann’s general relativity models cannot be extrapolated. We argued from Bekenstein’s universal entropy bound and the holographic bound taken to a time near enough to the Planck time where these bounds are contradicted, that there could not have been a Friedmann-type singularity.

Therefore, the above supports Bekenstein’s thermodynamically-based argument that the singularity cannot exist where the entropy bound is contradicted and relates it to the significance of entropy and information near the beginning following from Wheeler’s principle that everything is fundamentally information. Our proposed quantum particle horizon derived from the Bekenstein bound is a spacelike hypersurface that acts as a barrier to any extrapolations at earlier times. From Penrose, “Particle horizons occur in all standard cosmologies, arising from the past singularity being spacelike.” [25]. In Section 2, after Equation (6) (above), we calculated the apparent horizon based on backward null evolution from the observed time back to the initial classical singularity. However, we have seen that quantum gravity must inevitably become important, and that entropy becomes very small at some minimum time $t_{min}\sim t_{P}$. This is a boundary in time, temperature, and information beyond which we can go no further or know anything more about the beginning as in Penrose cosmic censorship. As Penrose states, “*The situation exhibited by the Big Bang … acting as a spacelike past boundary to the spacetime … The spacelike (hypersurface) nature of this initial boundary leads us to the notion of a particle horizon, which is an important aspect of the Big Bang*” (Italics ours) [25]. The microscopic details of this initial state are unknown, requiring a full theory of quantum gravity where quantum effects dominate gravitation, at the minimum irreducible radius of a quantum particle horizon, thus removing the classical inevitability of the cosmological singularity. Our approach parallels that of Bousso [22] and Kaloper and Linde [39], who proposed an ad hoc redefinition of the particle horizon as terminating or originating at $t\sim t_{P}$.

From “Wheeler’s Last Blackboard” [40]. “1. We don’t understand how the universe came into being …”. “16. The laws of physics reveal as little about the deeper structure of the universe as the laws of electricity reveal about the quantum mechanics of the solid state. Symmetry principles summarize law but also hide machinery behind the law …”. “Thus, it appears that we must confront the breakdown of classical general relativity expected to occur near singularities if we are to understand the origin of our universe” [41].
